# Supplementary material for: Primed to die: an investigation of the genetic mechanisms underlying noise-induced hearing loss and cochlear damage in homozygous Foxo3-knockout mice
Source: Cell Death Dis. 2021 Jul 7;12(7):682. doi: 10.1038/s41419-021-03972-6 (PMC8263610; doi:10.1038/s41419-021-03972-6)
Supplement: Supplementary file 1 — Supplemental Figures and Tables [file 41419_2021_3972_MOESM1_ESM.pdf]

Table S1. Primary antibodies used in this study.

Name	Primary Antibody (catalog no.)	Antibody Concentration	Company
Oncomodulin (OCM)	Polyclonal goat anti-OCM (sc-7446) - discontinued	1:500	Santa Cruz
	Polyclonal goat anti-oncomodulin (PA5-47832)	1:500	Thermo Fisher Sci
MYO7	Polyclonal rabbit anti-Myosin VIIa (sc-25834)	1:500	Santa Cruz
Cytochrome-C	Monoclonal mouse anti-Cytochrome c (556433)	1:500	BD Biosciences
CASP3	Monoclonal rabbit anti-cleaved caspase-3 (9664)	1:1000	Cell Signaling
AIFM1	Polyclonal rabbit anti-apoptosis-inducing factor 1-mitochondrial (NBP2-33932)	1:1000	Novus Biologicals
pJnk	Monoclonal rabbit anti-pJNK (4668)	1:250	Cell Signaling
pcJun	Monoclonal rabbit anti-Phospho-c-Jun (3270)	1:250	Cell Signaling
p53	Polyclonal rabbit anti-P53 (10442-1-AP)	1:500	Proteintech
4HNE	Monoclonal mouse anti-4-Hydroxynonenal (MAB3249)	1:500	R&D Systems
Hsp70	Polyclonal rabbit anti-HSP70 (4872)	1:200	Cell Signaling
GDPD3	Polyclonal rabbit anti-GDPD3 (PA5-59581)	1:50	Thermo Fisher Sci

Table S2. qPCR primer sequences used in this study.

Name	Forward Sequence	Reverse Sequence
Hprt	5’-TCAGTCAACGGGGGACATAAA-3’	5’-GGGGCTGTACTGCTTAACCAG-3’
GusB	5’-GGCTGGTGACCTACTGGATTT-3’	5’-GGCACTGGGAACCTGAAGT-3’
Junb	5’-TCACGACGACTCTTACGCAG-3’	5’-CCTTGAGACCCCGATAGGGA-3’
Gdpd3	5’-CTCTCCTGTACTTTGTTCTGCC-3’	5’-CCAGGCGGATAGGGAAGAC-3’

Figure S1.

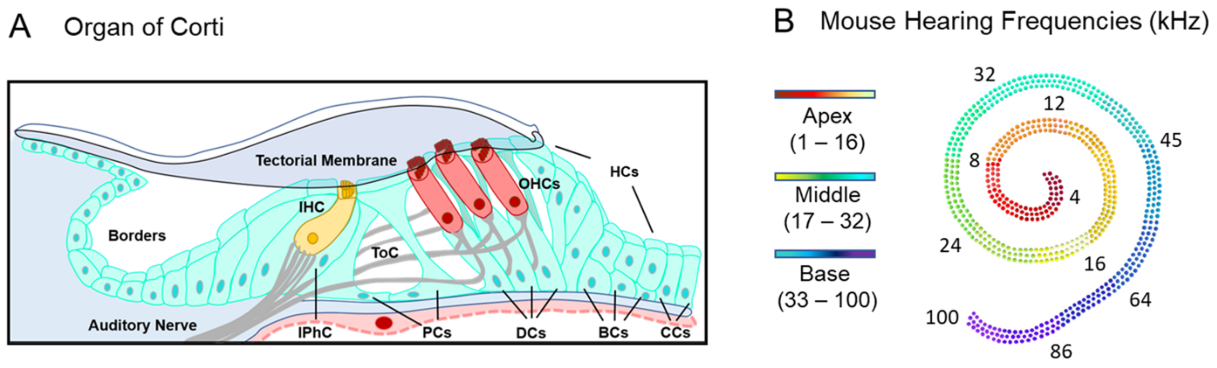

Supplementary Figure 1. Anatomy of the organ of Corti and mouse cochlear tonotopy. A) Schematic cross-section of the main cell populations within the organ of Corti. Sensory cells detect acoustic stimuli using stereocilia that bend due to tectorial membrane displacement and basilar membrane movement. Outer Hair Cells (OHCs, red) are arranged in three rows and act as amplifiers for acoustic signal transduction by one row of Inner Hair Cells (IHC, yellow). Efferent nerves, synapsing onto the OHCs and IHCs, and afferent nerves, synapsing onto the IHCs, bundle together to form the Auditory Nerve (gray). Several supporting cells aid in the homeostatic maintenance of the sensory cells surrounding the tunnel of Corti (ToC); these cells include the Border Cells (Borders), Inner Phalangeal Cell (IPhC), Pillar Cells (PCs), Deiters' Cells (DCs), Hensen's Cells (HCs), Boettcher Cells (BCs), and Claudius Cells (CCs) (cyan). This organization continues along the entire length of the cochlea. **B)** Diagram of estimated hearing frequencies in mouse cochlea. The mouse cochlea is arranged tonotopically and can detect frequencies up to 100 kHz. Low frequencies range from 1-16 kHz along the apex, middle frequencies from 17-32 kHz, and high frequencies from 33-100 kHz in the base.

Figure S2.

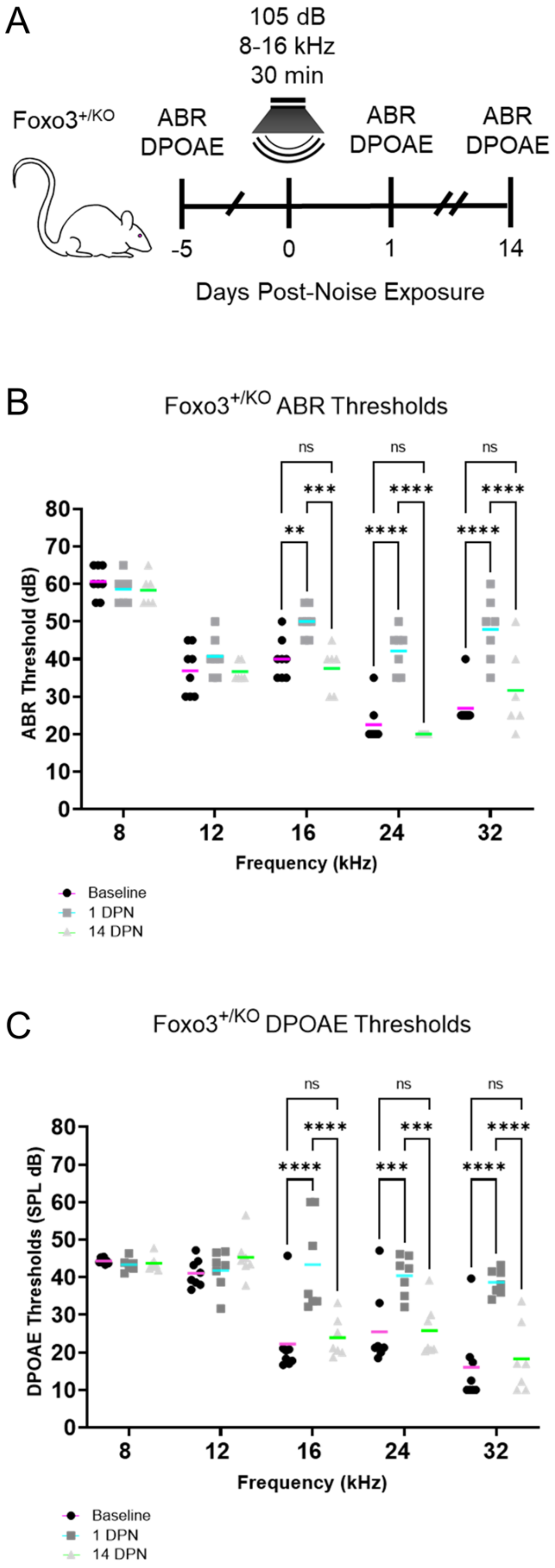
A
105 dB
8-16 kHz
30 min
Foxo3+/KO
ABR
DPOAE
ABR
DPOAE
ABR
DPOAE
-5
0
1
14
Days Post-Noise Exposure
B
Foxo3+/KO ABR Thresholds
ns
***
**
ns
****
****
ns
****
****
ABR Threshold (dB)
80
70
60
50
40
30
20
10
0
8
12
16
24
32
Frequency (kHz)
Baseline
1 DPN
14 DPN
C
Foxo3+/KO DPOAE Thresholds
ns
****
****
ns
***
***
ns
****
****
DPOAE Thresholds (SPL dB)
80
70
60
50
40
30
20
10
0
8
12
16
24
32
Frequency (kHz)
Baseline
1 DPN
14 DPN

Supplementary Figure 2. *Foxo3*$^{+/-}$ mice experience temporary threshold shifts after mild noise exposure. A) *Foxo3*$^{+/-}$ mice were subjected to an 8-16 kHz noise band at 105 dB for 30 min. Hearing tests were performed at baseline (-5 DPN), 1 DPN, and 14 DPN. **B)** ABR thresholds (y-axis) for individual *Foxo3*$^{+/-}$ mice are plotted along five frequencies tested (x-axis). Means are represented as bars for baseline (black circles, pink bars), 1 DPN (gray squares, cyan bars), and 14 DPN (light gray triangles, green bars). Significant threshold shifts were observed at 1 DPN at 16 kHz (**p = 0.0032), 24 kHz (****p < 0.0001) and 32 kHz (****p < 0.0001). ABR thresholds at these frequencies returned to baseline by 14 DPN. n = 6-8, two-way ANOVA with Bonferroni's multiple comparisons test, alpha = 0.05, *p < 0.05, **p < 0.01, ***p < 0.001, ****p < 0.0001. Females had better thresholds at 12 kHz (*p = 0.0266) but all other comparisons indicated similar threshold shifts and recovery between the sexes (2-3 males, 4-5 females, two-way ANOVA with Šidák's multiple comparisons test, alpha = 0.05, *p < 0.05). **C)** DPOAE thresholds with the same parameters as in **B**. Significant threshold shifts were observed at 1 DPN at 16 kHz (****p < 0.0001), 24 kHz (***p = 0.0003) and 32 kHz (****p < 0.0001). By 14 DPN at all frequencies, no significant differences were detected compared to baseline. n = 6-8, two-way ANOVA with Bonferroni's multiple comparisons test, alpha = 0.05, *p < 0.05, **p < 0.01, ***p < 0.001, ****p < 0.0001. Females had better 1 DPN thresholds at 16 kHz (*p = 0.0375) but no other significant differences were observed between the sexes (2-3 males, 4-5 females, two-way ANOVA with Šidák's multiple comparisons test, alpha = 0.05, *p < 0.05).

Figure S3.

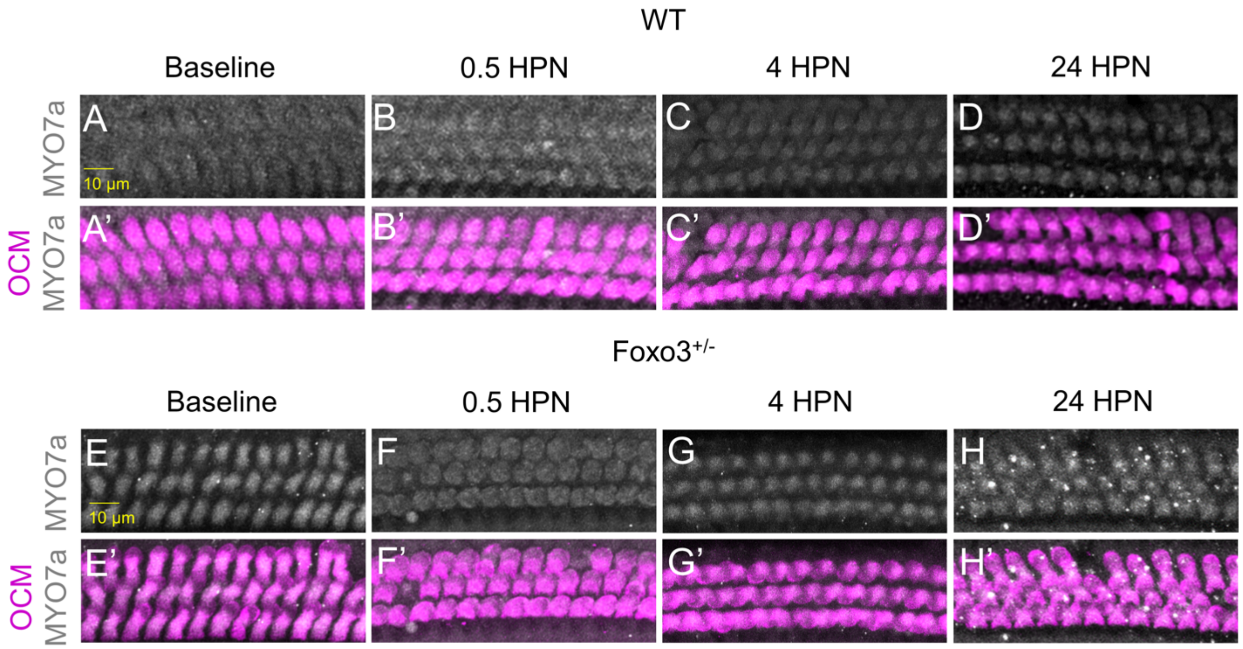

Supplementary Figure 3. WT and *Foxo3*$^{+/-}$ OHCs maintain MYO7a immunoreactivity at 24 kHz following noise exposure. Prior to and following noise exposure at 0.5, 4, and 24 hours, MYO7a expression (white) was maintained in the OHCs of WT **(A-D)** and *Foxo3*$^{+/-}$ **(E-H)** mice at the 24 kHz region. OCM (magenta) coexpressed with MYO7a in viable OHCs. Scale bar = 10 µm, n = 3 cochleae per genotype/condition.

Figure S4.

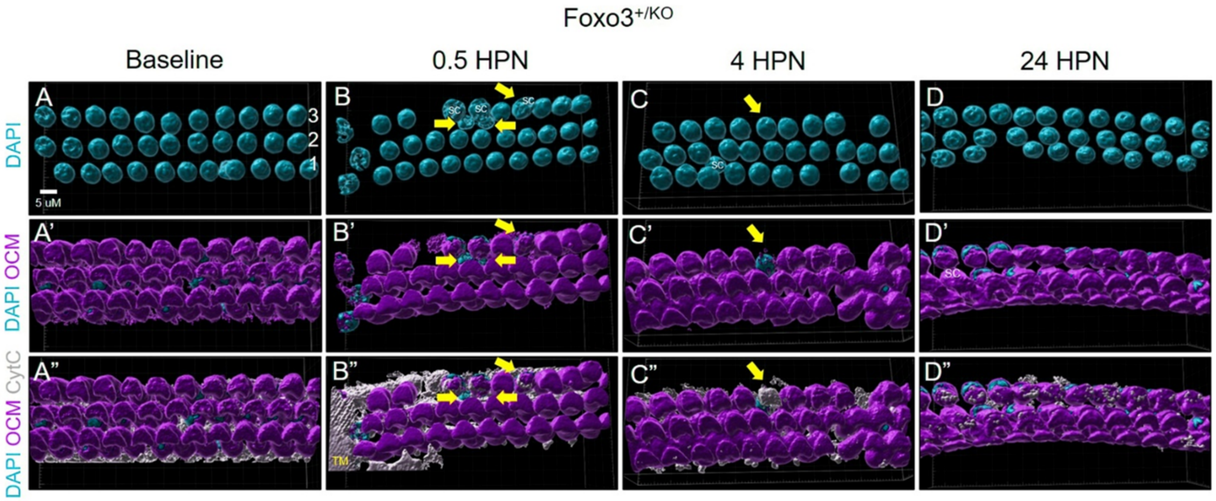

Supplementary Figure 4. At 24 kHz, *Foxo3*$^{+/-}$ mice show mild loss of OCM immunoreactivity following noise. After immunostaining in whole mount, the 24 kHz cochlear region was mapped, imaged using confocal microscopy at 150x magnification, and rendered in Imaris (n = 3 cochleae per genotype/condition). **A-D)** The rendered images of OHCs colored cyan for DAPI+ cell nuclei. **A'-D')** Renderings of DAPI+/OCM+ OHCs with OCM in magenta. **A"-D")** Renderings of DAPI+/OCM+/CytC+ cells with CytC in gray. n = 3 cochleae, scale bar = 10 µm, SC = supporting cell nuclei, TM = tectorial membrane. **B-B")** At 0.5 HPN, a minority of *Foxo3*$^{+/-}$ OHCs expressed less OCM immunoreactivity, mainly in rows 2 and 3 (yellow arrows). **C-C")** Only one OCM-/CytC+ OHC remained in row 3 by 4 HPN (yellow arrows). **D-D")** OCM expression and OHC numbers were comparable to controls.

62 **Figure S5.**

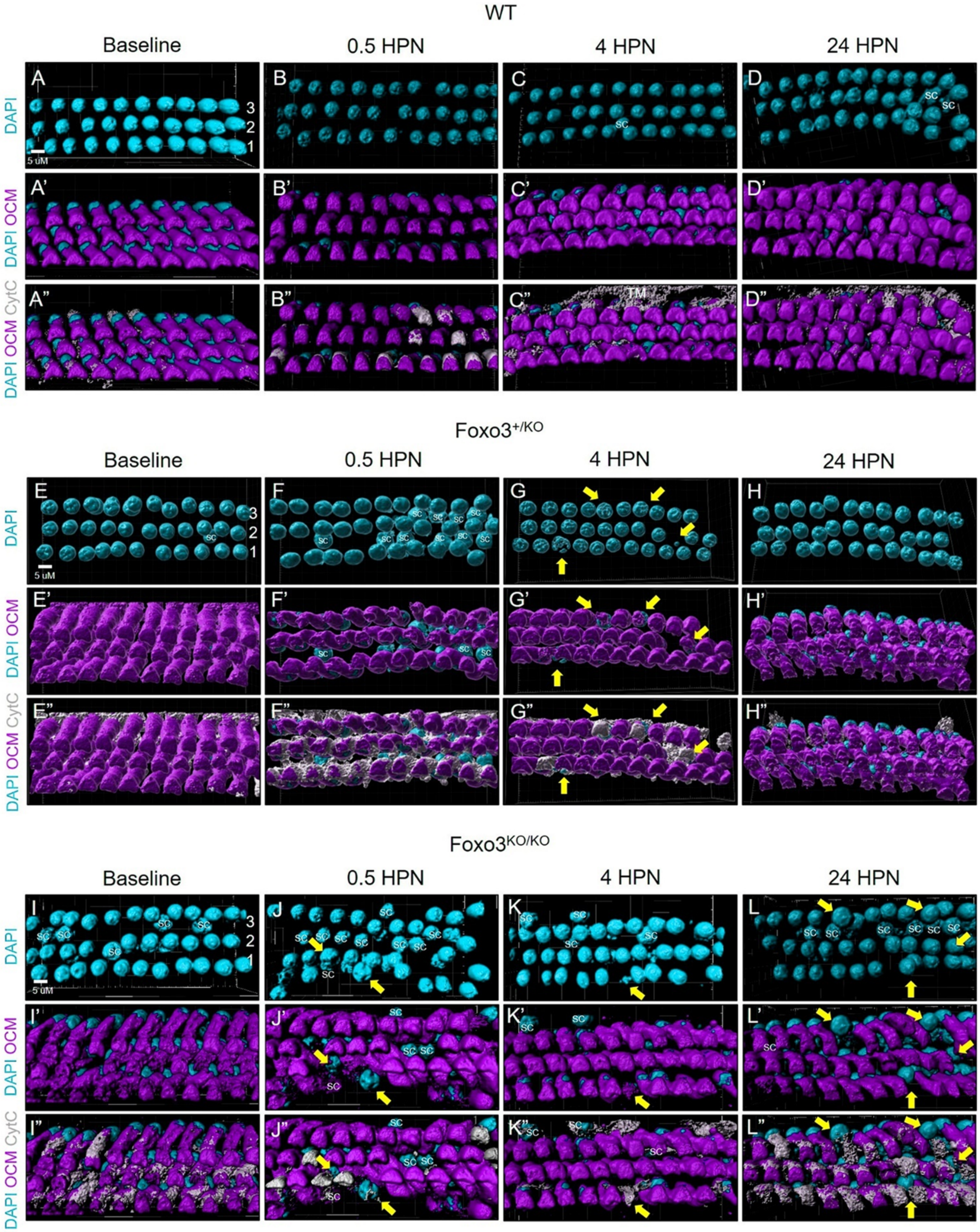

63

Supplementary Figure 5. Within the noise band, *Foxo3$^{-/-}$* OHCs undergo OCM modulation but little cellular loss by 24 HPN. Following whole mount histology, the 12 kHz cochlear region was mapped, imaged with confocal microscopy at 150x magnification, and rendered in Imaris (n = 3 cochleae per genotype/condition). **A-L)** The rendered images of OHCs DAPI+ cell nuclei in cyan. **A'-L')** Renderings of DAPI+/OCM+ OHCs with OCM in magenta. **A"-L")** Renderings of DAPI+/OCM+/CytC+ cells with CytC in gray. Scale bar = 10 µm, TM = remnants of tectorial membrane, SC = supporting cell nuclei. **A-D")** WT OHCs, **E-H")** *Foxo3$^{+/-}$* OHCs, **I-L")** *Foxo3$^{-/-}$* OHCs. **A-D")** WT OHCs showed no differences in OCM or CytC expression from baseline through 24 HPN. **E"-G")** *Foxo3$^{+/-}$* OHCs did not show strong changes in OCM immunoreactivity until 4 HPN (yellow arrows). **H-H"**) By 24 HPN, OCM expression was comparable to baseline levels in *Foxo3$^{+/-}$* OHCs. **J-J")** At 0.5 HPN, the *Foxo3$^{-/-}$* OHCs became disordered and several lost OCM immunoreactivity (yellow arrows). **K-K")** Pyknotic nuclei were present in OHCs with low OCM levels by 4 HPN (yellow arrow). **L-L")** OHCs without OCM showed slightly enlarged nuclei and thin cell bodies containing CytC (yellow arrows). **M-O)** OHC counts for all three genotypes expressing specific fluorophores (y-axis) were graphed versus the noise exposure timeline (x-axis). WT (black circles, orange mean bar), *Foxo3$^{+/-}$* (gray squares, pink mean bar), and *Foxo3$^{-/-}$* (light gray triangles, green mean bar) total cell counts per 1024 x 400 x 20-pixel selection. **M)** Numbers of DAPI+ OHC nuclei did not significantly differ between genotypes following noise. **N)** Counts of OHCs as calculated by totaling OCM+/CytC+ cells showed significant OCM modulation in *Foxo3$^{-/-}$* OHCs through 4 HPN. **O)** OHCs only expressing CytC increased in the *Foxo3$^{-/-}$* mice at 0.5 HPN but were comparable to baseline levels by 24 HPN. n = 3 per genotype/condition, two-way ANOVA with Tukey's test for multiple comparisons, alpha = 0.05, *p < 0.05, **p < 0.01, ***p < 0.001, ****p < 0.0001.

Figure S6.

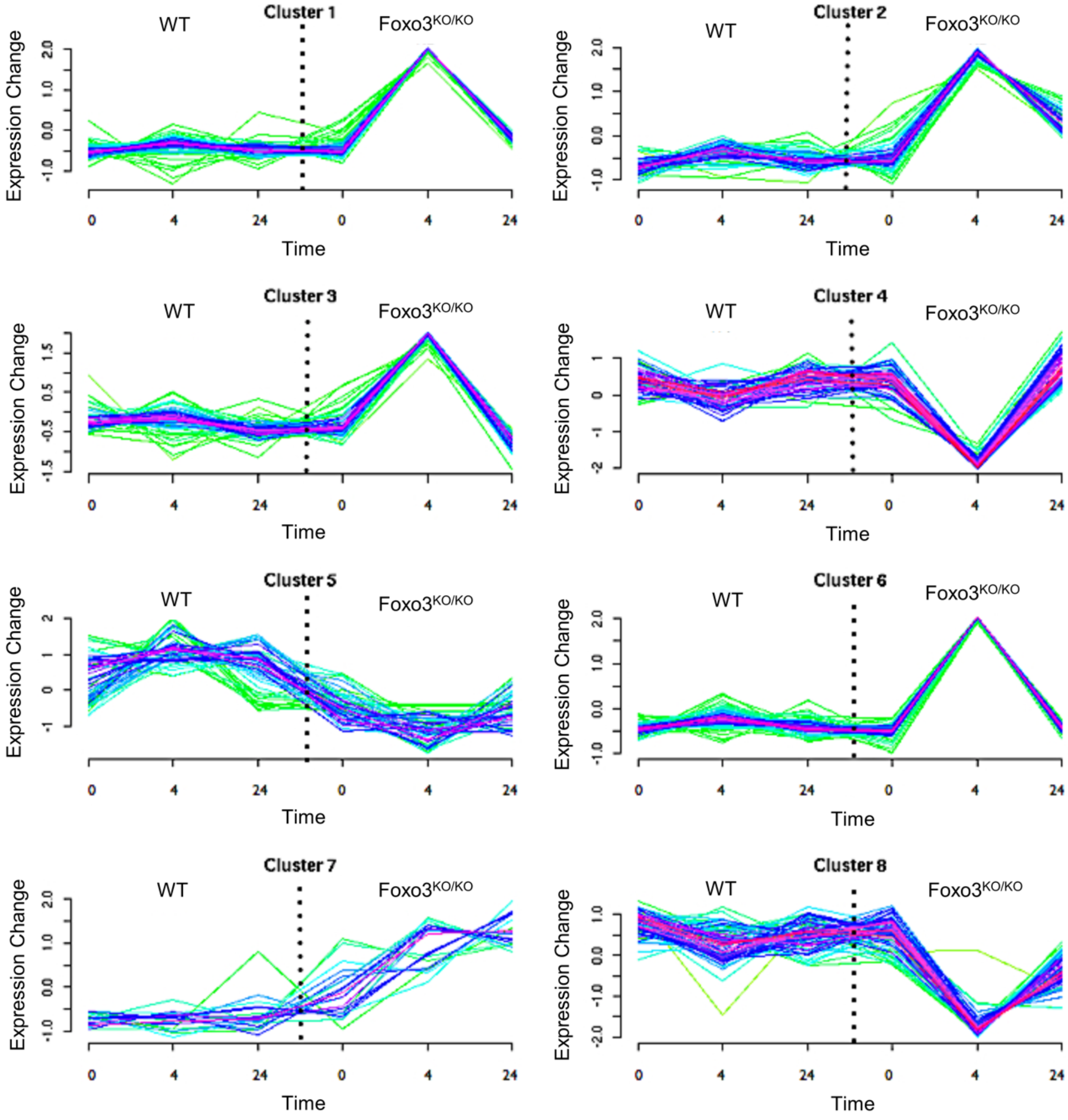

Supplementary Figure 6. Eight clusters of genes with similar expression change patterns observed between *Foxo3*$^{-/-}$ versus WT cochleae. Gene expression clustering was performed with Mfuzz for the *Foxo3*$^{-/-}$ versus WT cochleae at 4 HPN. DEGs had a fold change 2 threshold and false discovery rate (FDR) 5% correction. The resulting clusters contained genes with at least a 70% membership value. The expression rate (y-axis) is plotted against the time course of each

genotype (x-axis). Clusters 4, 5, and 8 showed decreases in gene transcript detection for the *Foxo3*$^{-/-}$ at 4 HPN.

Table S3.

Cluster 1		
**Genes**	**Membership**	**Function**
Atf3	72%	Stress responsive transcription factor
Bhlhe40	82%	Transcription factor that represses expression of Per, a circadian rhythm gene
Clcf1	81%	Potent neurotrophic factor that binds to the CNTF receptor
Fosl1	72%	Leucine zipper protein that dimerizes with Jun to form AP1
Sdc4	80%	Transmembrane receptor
Cluster 2		
**Genes**	**Membership**	**Function**
Bspry	73%	May regulate epithelial calcium transport by inhibiting TRPV5 activity
Epcam	84%	Homotypic calcium-independent cell adhesion molecule
Itga5	75%	Integrin alpha chain family
Krt18	86%	Encoded by RT18
Krt8	74%	Dimerizes with KRT18 to form an intermediate filament
Runx1	73%	Transcription factor that forms a complex with CBFB
Cluster 3		
**Genes**	**Membership**	**Function**
Bach1	72%	Transcriptional regulator that represses transcription of genes under the the NFE2L2 oxidative stress pathway
Ccnl1	80%	Involved in pre-mRNA splicing and functions in association with cyclin-dependent kinases (CDKs)
Fam107b	73%	Protein coding gene
Frat2	85%	Positive regulator of WNT signaling pathway
Hilpda	84%	Increases intracellular lipid accumulation and stimulates expression of cytokines including IL6, MIF and VEGFA
Nr4a3	82%	Transcriptional activator that can bind the NGFI-B Response Element (NBRE)
Nuak2	71%	Stress-activated kinase involved in tolerance to glucose starvation. Induces cell-cell detachment by increasing F-actin conversion to G-actin.
Pim1	76%	Involved in cell survival and proliferation; can phosphorylate and inhibit proapoptotic proteins BAD, MAP3K5, and FOXO3
Ppp1r15a	74%	Transcript levels are increased following stressful growth arrest conditions and treatment with DNA-damaging agents
Rnf19b	77%	E3 ubiquitin-protein ligase that plays a role in the cytotoxic effects of natural killer (NK) cells
Vcam1	71%	Mediates leukocyte-endothelial cell adhesion and signal transduction

Cluster 4		
**Genes**	**Membership**	**Function**
1700025G04Rik	91%	Unknown
2610017I09Rik	83%	Unknown
2610035F20Rik	84%	Unknown
4930402H24Rik	77%	Unknown
5031439G07Rik	72%	Unknown
Arhgap20	76%	Activator of RHO-type GTPases, transducing a signal from RAP1 to RHO and impacting neurite outgrowth
Bcat2	73%	Branched chain aminotransferase found in mitochondria
Ccdc141	72%	Radial migration and centrosomal function
Clmn	80%	Actin binding
Crot	80%	Helps transport medium- and long- chain acyl-CoA molecules out of the peroxisome to the cytosol and mitochondria
D630003M21Rik	82%	Unknown
Epn2	75%	Clathrin-mediated endocytosis and Notch Signaling Pathway
Esrrb	71%	Unknown but encodes a protein with similarity to the estrogen receptor
Fat2	85%	Cell adhesion molecule
G0s2	86%	Promotes apoptosis by binding to BCL2, preventing formation of protective BCL2-BAX heterodimers
Gprc5b	93%	GPCR that may modulate insulin secretion and increased protein expression
Itfg3	89%	Unknown
Met	72%	Prototypical receptor tyrosine kinase
Olfml1	71%	Unknown
Pdk1	85%	Protects cells against apoptosis in response to hypoxia and oxidative stress
Pou3f3	71%	Transcription factor that acts synergistically with SOX11 and SOX4
Ppp1r1b	72%	Dopaminergic and glutamatergic receptor stimulation regulates its phosphorylation and function as a kinase or phosphatase inhibitor
Slc4a11	73%	Involved in transport of potassium through the fibrocyte layer to the stria vascularis and is essential for the generation of the endocochlear potential but not for regulation of potassium concentrations in the endolymph
Sorcs3	72%	Type-I receptor transmembrane protein associated with Alzheimer's disease

Tcea3	81%	Necessary for efficient RNA polymerase II transcription elongation past template-encoded arresting sites
Tgfbr2	81%	Contributes to the transcription of genes related to cell proliferation, cell cycle arrest, and wound healing
Tmtc4	72%	TPR domains mediate protein-protein interactions in various cellular processes
Wnt5a	72%	Can activate or inhibit canonical Wnt signaling
Zic2	92%	Transcriptional repressor that may regulate tissue specific expression of dopamine receptor D1
Cluster 5		
**Genes**	**Membership**	**Function**
Igf1r	85%	Cell growth and survival control; can activate JNK kinases
Pgm2l1	74%	Intramolecular transferase activity, phosphotransferases and glucose-1,6-bisphosphate synthase activity
Sesn1	73%	Mediates p53 inhibition of cell growth by activating AMP-activated protein kinase, which results in the inhibition of the mammalian target of rapamycin protein
Syt9	70%	May be involved in Ca(2+)-dependent exocytosis of secretory vesicles through Ca(2+) and phospholipid binding to the C2 domain or may serve as Ca(2+) sensors in the process of vesicular trafficking and exocytosis
Tprgl	71%	Regulates synaptic release probability by decreasing the calcium sensitivity of release
Wdr45	81%	Component of the autophagy machinery that controls the major intracellular degradation process by which cytoplasmic materials are packaged into autophagosomes and delivered to lysosomes for degradation
Ypel3	80%	Involved in proliferation and apoptosis in myeloid precursor cells
Cluster 6		
**Genes**	**Membership**	**Function**
Csrnp1	72%	Has transcriptional activator activity and may play a role in apoptosis.
Dusp5	73%	Inactivates ERK1; negatively regulates members of the mitogen-activated protein (MAP) kinase superfamily (MAPK/ERK, SAPK/JNK, p38)
Egr1	82%	Regulates gene transcription for cell survival, proliferation and cell death; can activate expression of p53/TP53 and TGFB1
Fos	76%	Dimerizes with proteins of the JUN family to form the transcription factor complex AP-1.
Gadd45b	70%	Involved in the regulation of growth and apoptosis; mediates activation of stress-responsive MTK1/MEKK4 MAPKKK
Gdf15	88%	Increased protein levels are associated with disease states such as tissue hypoxia, inflammation, acute injury and oxidative stress
Gem	80%	Regulatory protein in receptor-mediated signal transduction
Hbegf	86%	Growth factor that mediates its effects via EGFR, ERBB2 and ERBB4
Junb	71%	Transcription factor involved in regulating gene activity following the primary growth factor response
Lif	80%	Pleiotropic cytokine
Maff	80%	Involved in the cellular stress response
Nfkbia	74%	Inhibits the activity of dimeric NF-kappa-B/REL complexes by trapping REL dimers in the cytoplasm through masking of their nuclear localization signals

Phlda 1	73%	Regulation of apoptosis
Plk3	73%	Rapidly activated upon stress stimulation, such as ionizing radiation, reactive oxygen species (ROS), hyperosmotic stress, UV irradiation and hypoxia.
Socs3	79%	Negative regulation of cytokines that signal through the JAK/STAT pathway
Cluster 7		
**Genes**	**Membership**	**Function**
Lamc 2	88%	Mediates the attachment, migration and organization of cells into tissues during embryonic development
Serinc 2	78%	Viral mRNA Translation and Metabolism
Socs2	72%	Regulates receptor turn-over; downregulates growth factors and upregualtes Trk receptors
Cluster 8		
**Genes**	**Membership**	**Function**
Aass	72%	Bifunctional enzyme that catalyzes the first two steps in lysine degradation
Ahcyl 2	72%	May be involved in the conversion of S-adenosyl-L-homocysteine to L-homocysteine and adenosine
Appl2	87%	Multifunctional adapter protein and master regulator
Crygn	73%	Member of the crystallin family of proteins that are localized to the refractive structure of vertebrate eye lenses
Gm19 990	76%	Unknown
Gpr30	81%	G-protein coupled estrogen receptor that binds to 17-beta-estradiol (E2) with high affinity, leading to rapid and transient activation of numerous intracellular signaling pathways
Ksr1	70%	Part of a multiprotein signaling complex which promotes phosphorylation of Raf family members and activation of downstream MAP kinases
Lctl	74%	Member of the glycosidase enzymes
Maf	77%	Acts as a transcriptional activator or repressor
Map2 k6	81%	Dual specificity protein kinase which acts as an essential component of the MAP kinase signal transduction pathway
Mapk 4	92%	Phosphorylates microtubule-associated protein 2 (MAP2) and MAPKAPK5
Megf9	91%	Unknown
Paqr8	87%	Unknown
Rab17	73%	Epithelial cell-specific GTPase
Rapge f3	72%	Guanine nucleotide exchange factor (GEF) for RAP1A and RAP2A small GTPases that is activated by binding cAMP
Slc18 b1	79%	Unknown
Slc8a 3	85%	Mediates the electrogenic exchange of Ca(2+) against Na(+) ions across the cell membrane, and thereby contributes to the regulation of cytoplasmic Ca(2+) levels and Ca(2+)-dependent cellular processes
Tprkb	85%	Component of the EKC/KEOPS complex

Tyr	71%	Catalyzes the initial and rate limiting step in the cascade of reactions leading to melanin production from tyrosine
Wdr91	87%	Functions as a negative regulator of the PI3 kinase/PI3K activity associated with endosomal membranes via BECN1, a core subunit of the PI3K complex

Supplementary Table 3. Gene clusters detected in comparison of *Foxo3*$^{-/-}$ versus WT cochleae at 4 HPN contain several stress response, calcium regulatory, and cytoskeletal factors. Composition of genes identified during clustering analysis, listed with their membership percentage and function adapted from the GeneCards human gene database.

Figure S7.

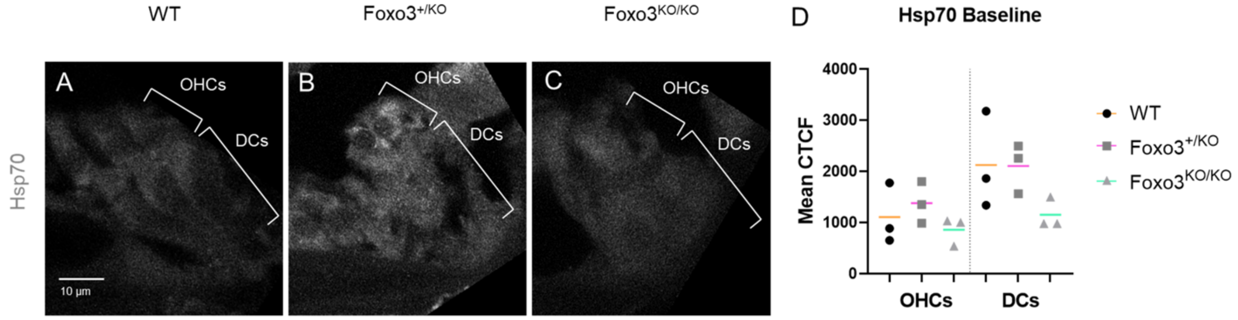

Supplementary Figure 7. No significant difference in Hsp70 expression within control OHCs and DCs between genotypes. A-C) Single-matched optical sections from confocal images of 24 kHz OHCs under control conditions. WT **(A)**, *Foxo3*$^{+/-}$ **(B)** and *Foxo3*$^{-/-}$ **(C)** cochleae were sectioned and immunostained for detection of Hsp70 (white) in OHCs and DCs. n = 3 per genotype, 60x magnification, scale bar = 10 µm. **D)** Quantification of the mean corrected total cell fluorescence (CTCF) of Hsp70 for OHCs and DCs. Each dot represents the CTCF means of each section and the bars are the means of those values: WT (black circles, orange mean bar), *Foxo3*$^{+/-}$ (gray squares, pink mean bar), and *Foxo3*$^{-/-}$ (light gray triangles, green mean bar). No significant differences in Hsp70 expression were observed between genotypes. Two-way ANOVA with Tukey's test for multiple comparisons, alpha = 0.05.

Figure S8.

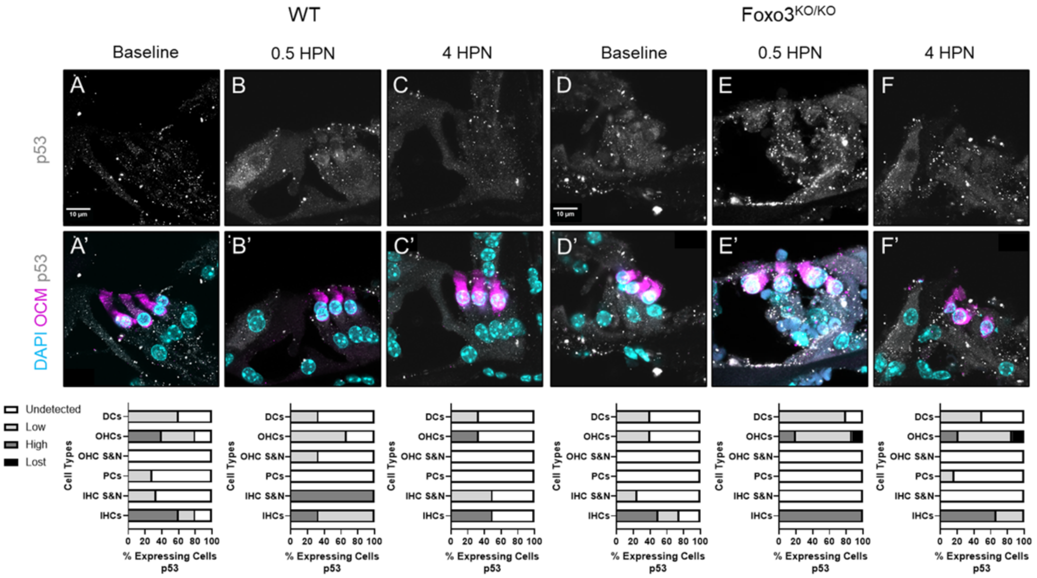

Supplementary Figure 8. Expression of p53 is not strongly modulated following noise exposure. WT and *Foxo3*$^{-/-}$ mice were exposed to noise and their cochleae were extracted for cryosectioning and immunohistochemistry at designated time points. **A-F')** Cochlear sections of the 24 kHz region immunostained for DAPI (cyan), OCM (magenta), and p53 (white) at baseline, 0.5 HPN, and 4 HPN. n = 3-5 per genotype/condition, 200x magnification, scale bar = 10 µm. Corresponding % fluorescent cellular expression graphs below each set of images: undetected = white, low = light gray, high = gray, and lost cells = black; DC: Deiters' Cells, OHCs: outer hair cells, OHC S&N: OHC synapses and neurites, PCs: pillar cells, IHC S&N: IHC synapses and neurites, and IHC: inner hair cells. With the exception of IHCs, weak p53 levels are detected in most cells of the organ of Corti with no strong changes seen in either genotype after noise.

Figure S9.

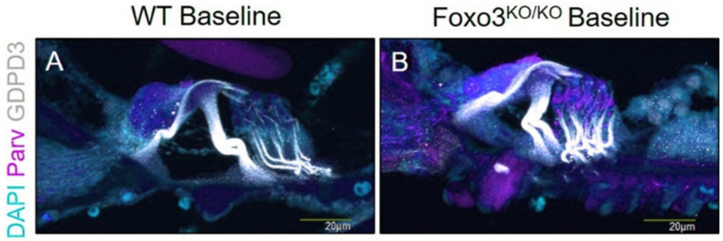

Figure S9. GDPD3 is expressed in cochlear supporting cells and neurites. A, B) Confocal images of GDPD3 (white) expressed in the pillar cells, Deiters' cells, and neurites of both WT and *Foxo3*$^{-/-}$ cochleae at baseline. DAPI (cyan), Parvalbumin (PARV, magenta), 24 kHz region, n = 3 per genotype, 60x magnification, scale bar = 20 µm.
